# The genome sequences of *Danio albolineatus* (Blyth, 1860), *Danio choprai* Hora, 1928, *Danio jaintianensis* (Sen, 2007) and *Danio tinwini* [Kullander & Fang], 2009

**DOI:** 10.12688/wellcomeopenres.25116.1

**Published:** 2025-11-20

**Authors:** Kerstin Howe, Braedan McCluskey, Uwe Irion, Ralf Britz, Shane A. McCarthy, Sarah Pelan, Jonathan M.D. Wood, Michelle Smith, Karen Oliver

**Affiliations:** 1 Wellcome Sanger Institute, Hinxton, England, UK; 2 Department of Biology, University of Virginia, Charlottesville, Virginia, USA; 3 Max Planck Insitute for Biology, Tübingen, Germany; 4 Department of Zoology, Natural History Museum, London, England, UK; 5 Department of Genetics, University of Cambridge, Cambridge, England, UK

**Keywords:** Danioninae, Danio albolineatus, Danio choprai, Danio jaintianensis and Danio tinwini, genome sequence, scaffold-level, Cypriniformes

## Abstract

We present the scaffold-level genome assemblies of four *Danio* species, released in 2020: *Danio albolineatus, Danio choprai, Danio jaintianensis* and *Danio tinwini* (Chordata; Actinopterygii; Cypriniformes; Cyprinidae). Genome sizes range from 1,102.16 Mb for *Danio choprai* to 1,497.36 Mb for *Danio tinwini*.

## Species taxonomy

*Danio tinwini* [Taxonomy ID: 1093597]:

Eukaryota; Metazoa; Chordata; Craniata; Vertebrata; Euteleostomi; Actinopterygii; Neopterygii; Teleostei; Ostariophysi; Cypriniformes; Danionidae; Danioninae; *Danio*; *Danio tinwini*

*Danio albolineatus* [Taxonomy ID: 27699]:

Eukaryota; Metazoa; Chordata; Craniata; Vertebrata; Euteleostomi; Actinopterygii; Neopterygii; Teleostei; Ostariophysi; Cypriniformes; Danionidae; Danioninae; *Danio*; *Danio albolineatus*

*Danio choprai* [Taxonomy ID: 299714]:

Eukaryota; Metazoa; Chordata; Craniata; Vertebrata; Euteleostomi; Actinopterygii; Neopterygii; Teleostei; Ostariophysi; Cypriniformes; Danionidae; Danioninae; Danio; Danio choprai

*Danio jaintianensis* [Taxonomy ID: 1982067]:

Eukaryota; Metazoa; Chordata; Craniata; Vertebrata; Euteleostomi; Actinopterygii; Neopterygii; Teleostei; Ostariophysi; Cypriniformes; Danionidae; Danioninae; Danio; Danio jaintianensis

## Background

The Danioninae are an emerging model clade in the field of evo-devo, favoured for analyses of anatomy (especially bone morphology and growth), pigmentation, gene family expansion and phylogeography ( Braasch *et al.*, 2015; Parichy, 2015). Within the Danioninae Sequencing Project (PRJEB38594) we have provided genome assemblies for representatives of this clade to facilitate its use. This includes the genome assemblies of five *Danio rerio* strains (Tuebingen, AB, SAT, Nadia and Cooch Behar) and eight additional species within the *Danio* and also the *Danionella* genus, the later representing anatomical extremes and a novel neuroscience model.

In this data note we present scaffold-level genome sequences of four *Danio* species – *D. tinwini*, *D. albolineatus*, *D. choprae* and *D. jaintianensis*. *D. tinwini* is endemic to upper Ayeyarwady (Irrawaddy) tributaries in northern Myanmar ( Fricke *et al.*, 2025d); *D. choprae* occurs in the Irrawaddy basin ( Fricke *et al.*, 2025b); *D. albolineatus* is widespread in mainland Southeast Asia ( Fricke *et al.*, 2025a); and *D. jaintianensis* is restricted to the Jaintia Hills of Meghalaya, India ( Fricke *et al.*, 2025c). These species have been used as comparative models for pigment pattern and colour biology – studies across *Danio* implicate endothelin signalling in pattern diversification, and work in *D. albolineatus* has resolved genetic determinants of red (erythrophore) pigmentation ( Spiewak *et al.*, 2018). Comparative transcriptomes also point to accelerated evolution of immune-related genes in *D. choprae* and *D. albolineatus*, underscoring their value for immunology ( Bao & Xia, 2017). Regional DNA-barcoding surveys in the Brahmaputra system provide additional context for north-east Indian *Danio* diversity relevant to *D. jaintianensis* ( Kundu *et al.*, 2019).

## Genome sequence report

**Figure 1.**
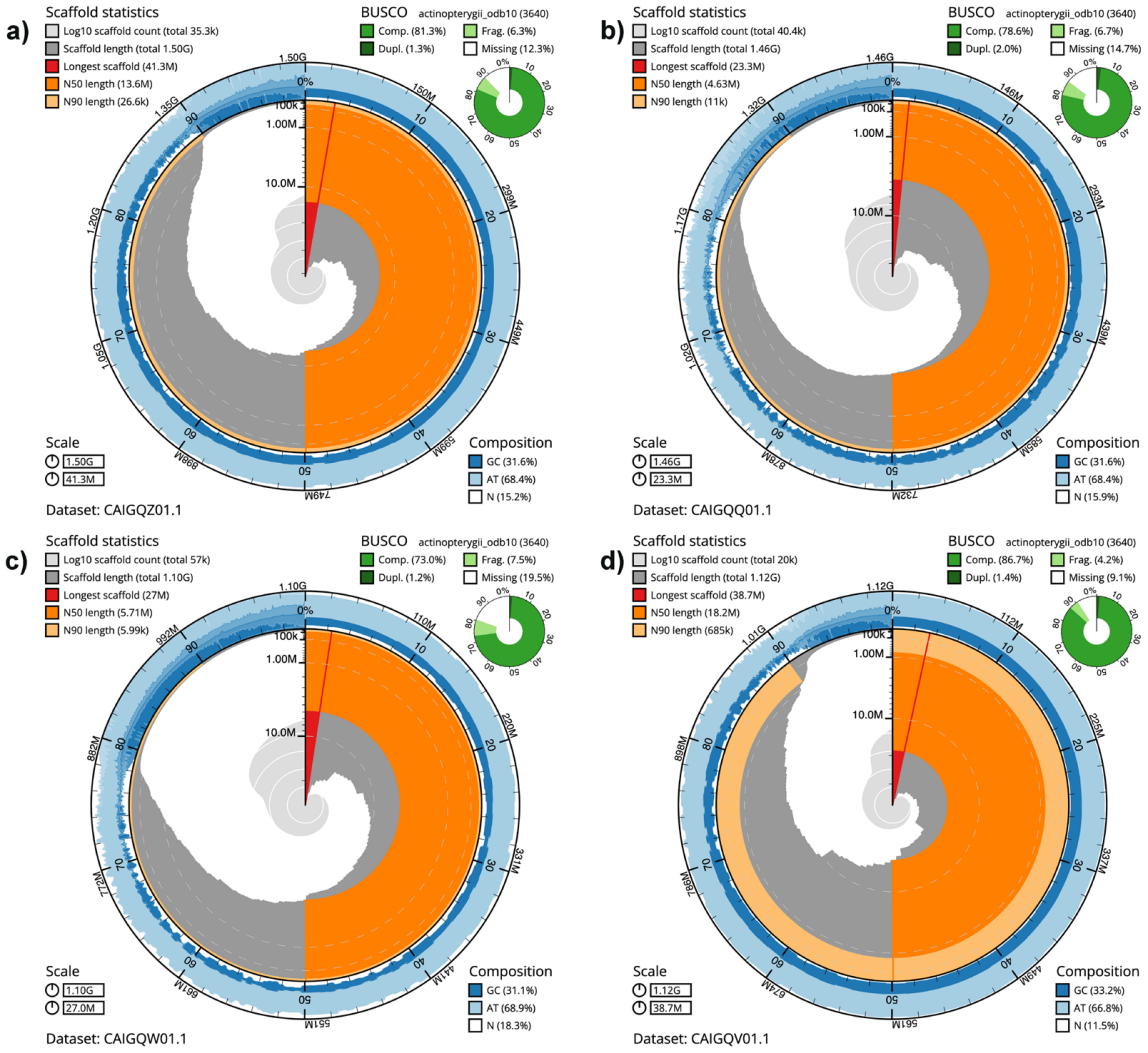
Genome assemblies of four *Danio* species: snail plots. **a**) *D. tinwini* **b**) *D. albolineatus* **c**) *D. choprai* **d**) *D. jaintianensis*. The BlobToolKit snail plots show N50 metrics and BUSCO gene completeness. The main plot is divided into 1,000 size-ordered bins around the circumference with each bin representing 0.1% of the assembly. The distribution of sequence lengths is shown in dark grey with the plot radius scaled to the longest sequence present in the assembly (shown in red). Orange and pale-orange arcs show the N50 and N90 sequence lengths, respectively. The pale grey spiral shows the cumulative sequence count on a log scale with white scale lines showing successive orders of magnitude. The blue and pale-blue area around the outside of the plot shows the distribution of GC, AT and N percentages in the same bins as the inner plot. A summary of complete, fragmented, duplicated and missing BUSCO genes in the actinopterygii_odb10 set is shown in the top right. The snail plots can be viewed interactively at the following links: fDanTin1, fDanAlb1, fDanCho1 and fDanJai1.

**Figure 2.**
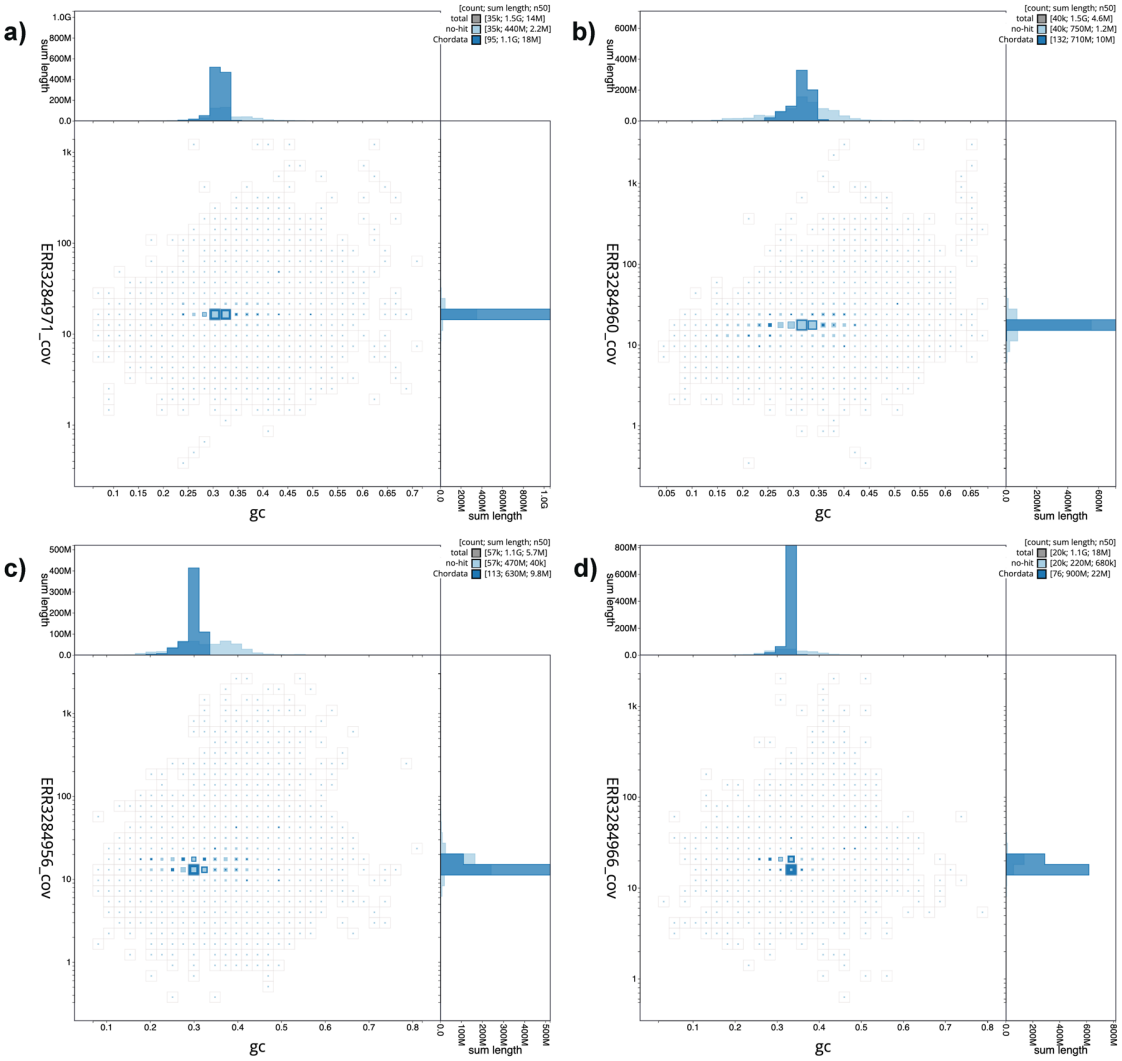
Genome assemblies of *Danio* species: BlobToolKit blob plots. **a**) *D. tinwini* **b**) *D. albolineatus* **c**) *D. choprai* **d**) *D. jaintianensis*. Scaffolds are coloured by phylum. Circles are sized in proportion to scaffold length. Histograms show the distribution of scaffold length sum along each axis. The blob plots can be viewed interactively at the following links: fDanTin1, fDanAlb1, fDanCho1 and fDanJai1.

**Figure 3.**
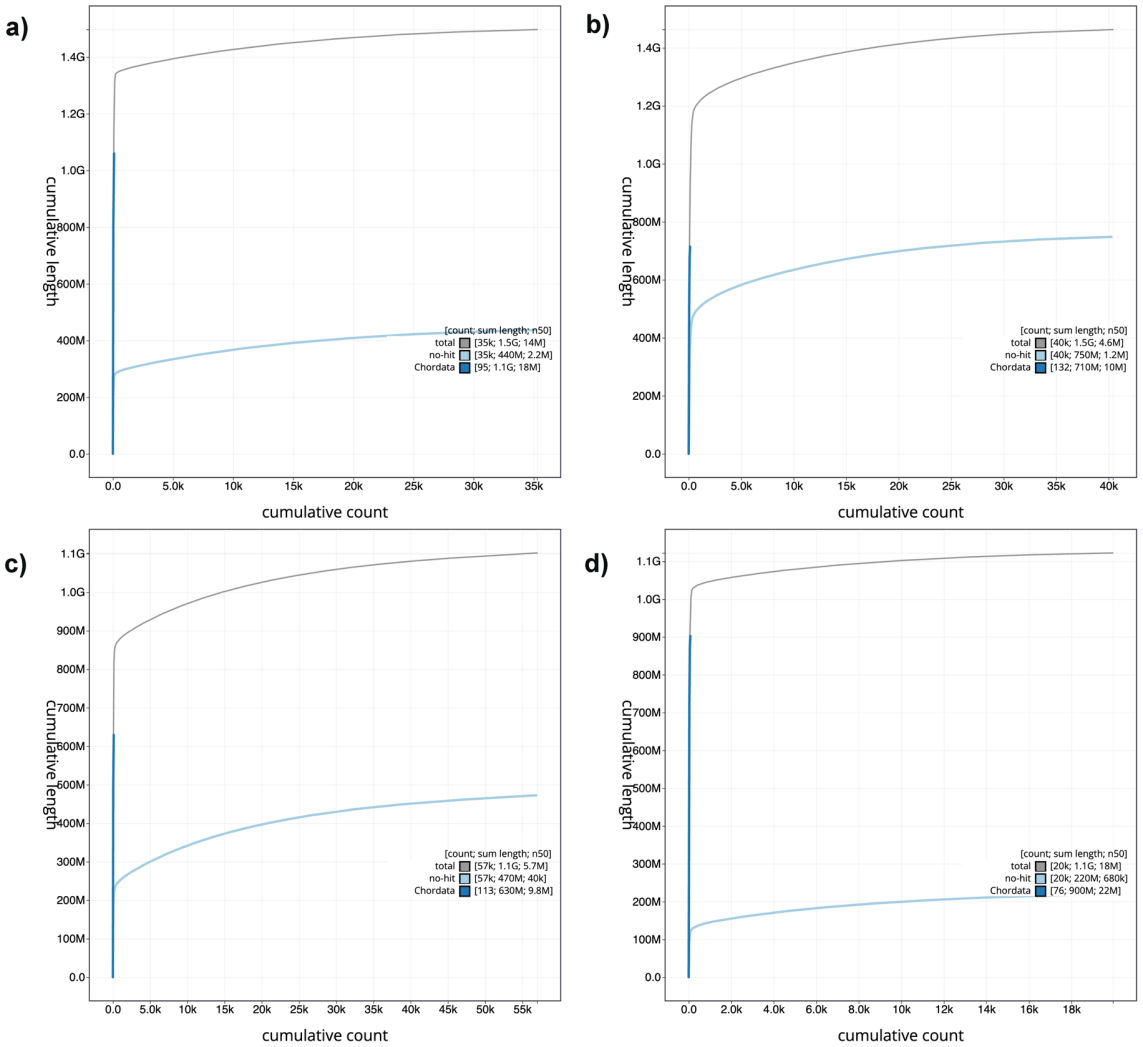
Genome assemblies of *Danio* species: BlobToolKit cumulative sequence plots. **a**) *D. tinwini* **b**) *D. albolineatus* **c**) *D. choprai* **d**) *D. jaintianensis*. The grey line shows cumulative length for all scaffolds. Coloured lines show cumulative lengths of scaffolds assigned to each phylum using the buscogenes taxrule. The cumulative plots can be viewed interactively at the following links: fDanTin1, fDanAlb1, fDanCho1 and fDanJai1.

Table 1 shows the statistics for each assembly. The snail plots in Figure 1 provide a summary of the assembly statistics, while the distribution of assembly scaffolds on GC proportion and coverage is shown in Figure 2. The cumulative assembly plots in Figure 3 show curves for subsets of scaffolds assigned to different phyla.

**Table 1.** Genome data for *Danio* species.

Project accession data				
Assembly identifiers	fDanTin1.1	fDanAlb1.1	fDanCho1.1	fDanJai1.1
Species	*Danio tinwini*	*Danio albolineatus*	*Danio choprai*	*Danio jaintianensis*
Specimen	fDanTin1	fDanAlb1	fDanCho1	fDanJai1
NCBI taxonomy ID	1093597	27699	299714	1982067
BioProject	PRJEB38572	PRJEB38566	PRJEB38568	PRJEB38569
BioSample ID	SAMEA104026413	SAMEA104026392	SAMEA104026429	SAMEA104026422
Raw data accessions	fDanTin1	fDanAlb1	fDanCho1	fDanJai1
10X Genomics Illumina	ERR3284969, ERR3284971, ERR3284970, ERR3284972	ERR3284958, ERR3284959, ERR3284957, ERR3284960	ERR3284953, ERR3284955, ERR3284956, ERR3284954	ERR3284965, ERR3284966, ERR3284968, ERR3284967
Genome assembly	fDanTin1.1	fDanAlb1.1	fDanCho1.1	fDanJai1.1
Assembly accession	GCA_903798205.1	GCA_903798035.1	GCA_903798125.1	GCA_903798115.1
Span (Mb)	1,497.36	1,463.02	1,102.16	1,122.50
Number of contigs	146,950	132,646	135,551	93,148
Contig N50 length (kb)	16.69	18.83	13.93	22.55
Number of scaffolds	35,277	40,406	56,986	19,951
Scaffold N50 length (Mb)	13.58	4.63	5.71	18.17
BUSCO completeness **	C:81.3%[S:80.0%,D:1.3%]	C:78.6%[S:76.6%,D:2.0%],	C:73.0%[S:71.8%,D:1.2%]	C:86.7%[S:85.3%,D:1.4%]

* BUSCO scores based on the actinopterygii_odb10 BUSCO set ( *n* = 3,640) using v5.3.2. C = complete [S = single copy, D = duplicated], n = number of orthologues in comparison. Full sets of BUSCO scores are available for fDanTin1, fDanAlb1, fDanCho1 and fDanJai1.

The *Danio tinwini* (fDanTin1.1) assembly has a BUSCO completeness of 81.3% (single = 80.0%, duplicated = 1.3%).

The *Danio albolineatus* (fDanAlb1.1) assembly has a BUSCO completeness of 78.6% (single = 76.6%, duplicated = 2.0%).

The *Danio choprai* (fDanCho1.1) assembly has a BUSCO completeness of 73.0% (single = 71.8%, duplicated = 1.2%).

The *Danio jaintianensis* (fDanJai1.1) assembly has a BUSCO completeness of 86.7% (single = 85.3%, duplicated = 1.4%).

For all BUSCO analyses, BUSCO version 5.3.2 was used with the actinopterygii_odb10 reference set ( *n* = 3,640).

## Methods

### Sample acquisition and nucleic acid extraction

The *Danio albolineatus* and *Danio choprai* samples were provided by Uwe Irion, MPI for Developmental Biology, Tuebingen. The *Danio jaintianensis* sample was provided by Ralf Britz, Natural History Museum, London. The *Danio tinwini* sample was provided by Braedan McCluskey, University of Virginia.

Nucleic acid extraction was carried out using Bionano Prep Cell Culture DNA Isolation Protocol. In this method, the cells are first embedded in agarose to provide structural support during the extraction process. The agarose-embedded cells are then treated with lysis buffers to break down the cell membranes and release the DNA. The process also involves proteinase digestion to remove proteins, followed by a series of washes to purify the DNA.

### Sequencing

10X Genomics read cloud DNA sequencing libraries were constructed according to the manufacturers’ instructions. Sequencing was carried out at the Scientific Operations Core at the Wellcome Sanger Institute on an Illumina HiSeq X Ten instrument.

### Genome assembly, curation and evaluation

The four assemblies were generated using 10X Genomics Chromium data. The fold coverage for each assembly was estimated as 70x for fDanAlb1.1, 57x for fDanCho1.1, 85x for fDanJai1.1, and 72x for fDanTin1.1. In each case, the assembly process included initial assembly generation using Supernova 2.0.1 and retained haplotig identification with Purge Haplotigs ( Roach *et al.*, 2018). Finally, the assembly was analysed and manually improved using gEVAL ( Chow *et al.*, 2016).

The genome was analysed within the BlobToolKit environment ( Challis *et al.*, 2020) and BUSCO scores ( Manni *et al.*, 2021) were calculated.

Table 2 contains a list of relevant software tool versions and sources.

**Table 2.** Software tools: versions and sources.

Software tool	Version	Source
BlobToolKit	4.2.1	https://github.com/blobtoolkit/blobtoolkit
BUSCO	5.3.2	https://gitlab.com/ezlab/busco
gEVAL	2016	https://geval.org.uk/
Purge haplotigs	-	https://bitbucket.org/mroachawri/purge_haplotigs
Supernova	2.0.1	https://github.com/10XGenomics/supernova

### Wellcome Sanger Institute – Legal and Governance

The materials that have contributed to this genome note have been supplied by a Tree of Life collaborator. The Wellcome Sanger Institute employs a process whereby due diligence is carried out proportionate to the nature of the materials themselves, and the circumstances under which they have been/are to be collected and provided for use. The purpose of this is to address and mitigate any potential legal and/or ethical implications of receipt and use of the materials as part of the research project, and to ensure that in doing so we align with best practice wherever possible.

The overarching areas of consideration are:

•    Ethical review of provenance and sourcing of the material

•    Legality of collection, transfer and use (national and international)

Each transfer of samples is undertaken according to a Research Collaboration Agreement or Material Transfer Agreement entered into by the Tree of Life collaborator, Genome Research Limited (operating as the Wellcome Sanger Institute) and in some circumstances other Tree of Life collaborators.

## Data Availability

Data has been deposited at the European Nucleotide Archive under the BioProjects for *Danio albolineatus* ( PRJEB38566), *Danio choprai* ( PRJEB38568), *Danio jaintianensis* ( PRJEB38570) and *Danio tinwini* ( PRJEB38572). The genome sequences are released openly for reuse. The *Danio* species genome sequencing initiative is part of the Danioninae Sequencing Project and the Vertebrate Genomes Project. All raw sequence data and the assembly have been deposited in INSDC databases. Raw data and assembly accession identifiers are reported in Table 1.
