# Supplementary material for: The genome sequences of Danio albolineatus (Blyth, 1860), Danio choprai Hora, 1928, Danio jaintianensis (Sen, 2007) and Danio tinwini [Kullander & Fang], 2009
Source: Wellcome Open Res. 2025 Nov 20;10:642. [Version 1] doi: 10.12688/wellcomeopenres.25116.1 (PMC12775661; doi:10.12688/wellcomeopenres.25116.1)
Supplement: Peer review history

# Reviewer report: Reviewer response for version 1

**DOI:** 10.21956/wellcomeopenres.27689.r142599

**Published:** 2026-01-06

**Authors:** Ivan Rodrigo Wolf

**Affiliations:** 1 Biological Sciences, UNC Charlotte Department of Biological Sciences (Ringgold ID: 124549), Charlotte, North Carolina, USA; 2 Universidade Estadual Paulista, São Paulo,, Brazil

The manuscript presents the assembly of four Danio species: *Danio albolineatus*, *Danio choprai*, *Danio jaintianensis* and *Danio tinwini*. these species can be found in a wide geographical areas in Myanmar ( *D. tinwini* and *D. choprae*), mainland Southeast Asia ( *D. albolineatus*) and India ( *D. jaintianensis*) and are being used as model organisms for research in fish diversity, pigmentation and comparative immunology.

 Biomolecule extraction and sequencing are adequate for the purpose of genomic assembly. Coverage and assembly procedures are as expected for the data obtained. The percentages of genes identified by BUSCO being below 90% appear to be influenced by the absence of complete chromosomal scaffolds.

 Considering the lack of more genomic information for the species analyzed so far, these genomes are an important resource for comparative genomics and variability and evolutionary analyses.

Are sufficient details of methods and materials provided to allow replication by others?

Yes

Is the rationale for creating the dataset(s) clearly described?

Yes

Are the datasets clearly presented in a useable and accessible format?

Yes

Are the protocols appropriate and is the work technically sound?

Yes

Reviewer Expertise:

Bioinformatics, systems biology, genomics, transcriptomics, molecular phylogenetics, immunogenomics, pattern recognition, search and characterization of genes of biotechnological interest.

I confirm that I have read this submission and believe that I have an appropriate level of expertise to confirm that it is of an acceptable scientific standard.

# Reviewer report: Reviewer response for version 1

**DOI:** 10.21956/wellcomeopenres.27689.r143209

**Published:** 2026-01-06

**Authors:** Alan Elena

**Affiliations:** 1 Technische Universität Dresden, Dresden, Germany

Howe *et al.* announce the availability of genomes belonging to four different *Danio* species. Authors briefly mention their motivation and provide quality data and accession numbers for public access. Even if the nature of this manuscript is to provide potential users with the source of relevant genomic sequences, I feel there are some points the authors should revise:

 1. PRJEB38572 is an umbrella project that contains the bioproject PRJEB38571. Is this for a reason? Otherwise, I feel it can be confusing as it may suggest that these are two different sets of sequencing results. The same is also true for the other bioprojects.

 2. Any discussion as to why this is the only available assembly would be nice. Is it because of technical challenges or because of low interest in these species. In any case, I think it would be beneficial if authors could briefly mention the implications of their contributions.

 3. The authors state the importance of the *Danio* genus as model organisms for anatomy, pigmentation and phylogeography. They also mention that they have previously contributed to this field by sequencing and assembling different *Danio* species, with this in mind: Are all *Danio* species equally relevant as model organisms?

 4. Based on the completeness values reported there seems to be between 14 and 27% of missing data in the assemblies. I believe that missing roughly a quarter of a genome is an important issue to address, specially if the provided assemblies are intended to serve as reference sequences for model organisms. Do the authors know what is missing from the provided sequences? Maybe this completeness issue can be improved by sequencing more than one representative of each species and converging them into a consensus genome. Missing data would be there due to the nature of building a consensus but still, the core genome and all the relevant sequences for different studies would be there.

 5. Figure 1 and 2 would benefit from having the text enlarged, specially axes and legends. In figure 2, the description mentions that the circle size represents scaffold length, due to the size, it is quite difficult to assess size differences.

 6. What does figure 3 means? Why is this figure in the “methods” section? I would suggest the authors to explain something about it. As it is, it looks like the figure was placed there in a draft and not moved around in the final version.

 7. Even if this is a report of availability of certain sequences, I think some discussion would greatly contribute to the quality of the work, for example:

1. Which is the impact of having such assemblies available?
2. Why are the provided sequences so incomplete? If so, does this have an effect for users that might use these sequences (See comment 4)
3. What does the quality data say about the overall quality of the presented sequences? Are these reliable? To which extent?

8. Regarding the methods, I feel some additional detail is needed:

- Which tissue is the origin of the recovered cells?
- How were these cells harvested?
- The DNA extraction protocol is rather vague. Please describe it in more detail or cite the corresponding bibliography.
- Which kind of sequencing was carried out? Was it short read?

9. **Major:** Regarding the sequencing technology, if a short-read approach was applied, I would suggest the authors to reassess the genome by carrying out a long read approach and a subsequent hybrid assembly. Short read assemblies are not enough, specially in superior organisms, to reliably map the genome. This is an even more concerning topic if the provided sequences are listed as reference sequences.

Are sufficient details of methods and materials provided to allow replication by others?

No

Is the rationale for creating the dataset(s) clearly described?

Partly

Are the datasets clearly presented in a useable and accessible format?

Yes

Are the protocols appropriate and is the work technically sound?

Partly

Reviewer Expertise:

Microbiology, bioinformatics

I confirm that I have read this submission and believe that I have an appropriate level of expertise to confirm that it is of an acceptable scientific standard, however I have significant reservations, as outlined above.

# Reviewer report: Reviewer response for version 1

**DOI:** 10.21956/wellcomeopenres.27689.r142193

**Published:** 2025-12-30

**Authors:** Bingpeng Xing

**Affiliations:** 1 Third Institute of Oceanography Ministry of Natural Resources, Xiamen, Fujian, China

1 It would be useful to add a short sentence to the Abstract, such as:

 “These assemblies provide scaffold-level genomic resources for comparative studies within Danioninae.”

 2 The Background section outlines the relevance of Danioninae as an emerging model clade, but the rationale for presenting scaffold-level genomes of these four species as a separate data resource is not entirely clear. In particular, the manuscript would benefit from a clearer positioning of these assemblies relative to previously published genomes of Danio rerio and other Danio species, especially with respect to assembly level (chromosome-level versus scaffold-level).

 3 BUSCO completeness values are moderate overall (73.0%–86.7%), with D. choprai showing the lowest completeness. The manuscript does not currently offer any explanation or technical context for these relatively low BUSCO scores.

 4 The Results section largely consists of a presentation of assembly statistics, with limited descriptive or interpretive text. Differences in assembly quality among the four species are not discussed, and the wide range in scaffold N50 values (4.63–18.17 Mb) is not commented on.

 5 The manuscript does not include a Discussion section. While a full discussion is not mandatory for a Data Note, adding a brief paragraph addressing the intended use of the data and its limitations would improve clarity and is increasingly common in genome resource publications.

Are sufficient details of methods and materials provided to allow replication by others?

Yes

Is the rationale for creating the dataset(s) clearly described?

Yes

Are the datasets clearly presented in a useable and accessible format?

Partly

Are the protocols appropriate and is the work technically sound?

Yes

Reviewer Expertise:

marine biology，genome，biodiversity，mitochandrial genome

I confirm that I have read this submission and believe that I have an appropriate level of expertise to confirm that it is of an acceptable scientific standard.
